# Coumarin compounds of *Biebersteinia multifida* roots show potential anxiolytic effects in mice

**DOI:** 10.1186/2008-2231-21-51

**Published:** 2013-06-27

**Authors:** Hamid Reza Monsef-Esfahani, Mohsen Amini, Navid Goodarzi, Fatemeh Saiedmohammadi, Reza Hajiaghaee, Mohammad Ali Faramarzi, Zahra Tofighi, Mohammad Hossein Ghahremani

**Affiliations:** 1 Department of Pharmacognosy, Faculty of Pharmacy, Tehran University of Medical Sciences, Tehran, Iran; 2 Department of Medicinal Chemistry, Faculty of Pharmacy, Tehran University of Medical Sciences, Tehran, Iran; 3 Department of Pharmaceutics, Faculty of Pharmacy, Tehran University of Medical Sciences, Tehran, Iran; 4 Nanotechnology Research Centre, Faculty of Pharmacy, Tehran University of Medical Sciences, Tehran, Iran; 5 Department of Pharmacology and Toxicology, Faculty of Pharmacy, Tehran University of Medical Sciences, Tehran, Iran; 6 Pharmacognosy & Pharmaceutics Department of Medicinal Plants Research Center, Institute of Medicinal Plants, ACECR, Karaj, Iran; 7 Department of Pharmaceutical Biotechnology, Faculty of Pharmacy & Biotechnology Research Center, Tehran University of Medical Sciences, Tehran, Iran

**Keywords:** Biebersteinia multifida, Coumarin, Anxiolytic, Scopoletin, Umbelliferone

## Abstract

**Background:**

Traditional preparations of the root of *Biebersteinia multifida* DC (Geraniaceae), a native medicinal plant of Irano-Turanian floristic region, have been used for the treatment of phobias as anxiolytic herbal preparation.

**Methods:**

We utilized the phobic behavior of mice in an elevated plus-maze as a model to evaluate the anxiolytic effect of the plant extract and bio-guided fractionation was applied to isolate the active compounds. Total root extract, alkaline and ether fraction were administered to mice at different doses 30 and 90 min prior to the maze test. Saline and diazepam were administered as negative and positive controls, respectively. The time spent in open and closed arms, an index of anxiety behavior and entry time, was measured as an index of animal activity.

**Results:**

The total root extract exhibited anxiolytic effect which was comparable to diazepam but with longer duration. This sustained effect of the crude extract was sustained for 90 min and was even more after injection of 45 mg/kg while the effect of diazepam had been reduced by 90 min. The anxiolytic effect factor was only present in the alkaline fraction and displayed its effect at lower doses than diazepam while pure vasicinone as the previously known alkaloid did not shown anxiolytic effect. The effect of the alkaline fraction was in a dose dependent manner starting at 0.2 mg/kg with a maximum at 1.0 mg/kg. Bio-guided fractionation using a variety of chromatographic methods led to isolation and purification of three coumarin derivatives from the bioactive fraction, including umbelliferone, scopoletin, and ferulic acid.

**Conclusion:**

For the first time, bio-guided fractionation of the root extract of *B. multifida* indicates significant sustained anxiolytic effects which led to isolation of three coumarin derivatives with well-known potent MAO inhibitory and anti-anxiety effects. These data contribute to evidence-based traditional use of *B. multifida* root for anxiety disorders.

## Background

*Biebersteinia multifida* DC (Geraniaceae), a native plant of Irano-Turanian floristic regions [1], is known traditionally as Chelleh-Dagh or Adamak in Iran. All four species of Biebersteinia distributed geographically from central Asia to Greek in temperate mountain zones. Among these pharmacologically active species, only *B. multifida* and *B. orphanidis* have tuberous roots. In folk medicine, the tuberous roots of *B. multifida* have been used topically for the relief of inflammation and pain of musculoskeletal disorders [2,3] and orally in the treatment of nocturia in children and of phobia and anxiety in humans and domestic animals [4] with no systematic approach to characterize the observed ethnopharmacological effects. Thus far, isolation of an alkaloid, vasicinone, and number of polysaccharides and peptide substances has been reported [5]. Flavonoids including 7-glucosides of apigenin, luteolin, and tricetin, as well as the 7-rutinoside of apigenin and luteolin have been isolated from its leaves which in part are responsible for antioxidant and antihemolytic activities [6-8]. Recently, essential oil composition of *B. multifida* was studied which exhibited that the main components were (E)-nerolidol, phytol, 6,10,14-trimethyl-2- pentadecanone and hexadecanoic acid [9].

Ethnopharmacological studies have revealed that the root extract of this plant has anti-inflammatory and analgesic activities that confirm the traditional use of *B. multifida* for the treatment of joint disturbances as well as in restoring bone fractures [10]. However, no report has yet been made on the anti-anxiety effects of the plant.

The elevated plus-maze has been developed as an ethological model of provoked anxiety and its use for the study of animal anxiety-like behavior has been pharmacologically validated and widely used for rats and mice [11-13]. In the present study, we have utilized the phobia behavior of mice in the elevated plus-maze as a model to evaluate the anxiolytic effect of the plant. For this purpose, the anxiolytic effects of the total root extract of *B. multifida* and its fractions were evaluated in mice and the chemical composition of the active fraction was identified.

## Materials and methods

### Chemicals

All chemicals were obtained from Merck (Darmstadt, Germany). Solvents used in chromatography methods were HPLC-grade. Diazepam (purity: not less than 98.0%) was obtained from Chemidarou Pharmaceutical Company (Tehran, Iran). Vasicinone (purity ≥97%) was a generous gift from Dr. Vahid Ziaee in Department of Medicinal Chemistry, Tehran University of Medical Sciences.

### Plant materials

The plant materials were collected from the region of Ruine, located in North Khorasan Province of Iran following the national rule on biodiversity by local agent of Iran Department of Environment. A voucher specimen has been deposited at the Herbarium of the Faculty of Pharmacy, Tehran University of Medical Sciences (Voucher No. 6691 TEH) by Prof. GR Amin. The root of the plant was used in this study.

### Chromatographic apparatus

A high performance liquid chromatography (HPLC) instrument equipped with K-1001 pump (Knauer, Germany), a D-14163 manual injector valve (Rheodyne), and a K-2600 UV detector for peak detection was used in the analytical studies. Another HPLC instrument (Knauer, Germany) including preparative K-1800 pumps (Double), two switching valves and a K-2501 UV detector were used for preparative purification. Solvents were filtered through a Millipore system and separation was performed on Knauer Eurosphere 100 C18 columns (150 mm × 4.0 mm, I.D. 5 μm) and (120 mm × 16 mm, I.D. 5 μm) for analytical and preparative HPLC, respectively.

### Spectroscopy instruments

The ^1^H-NMR and ^13^C-NMR spectra of the isolated compounds were measured in DMSO-*d*_*6*_ or CDCl_3_ at 500 and 125 MHz, respectively, using a Bruker AC 500 spectrophotometer (Germany). Mass spectra were prepared on Finnigan-Mat TSQ-70 spectrometer (CA, USA). Fourier-transform infrared (FTIR) spectra were obtained with a Nicolet Magna-FTIR 550 spectrometer (WI, USA).

### Extraction procedures

The roots were collected from the field, cleaned, dried for two weeks in the shade, then powdered and stored in airtight containers. The total extract was prepared from the powdered root refluxed with methanol for 72 hours using a Soxhlet apparatus, followed by filtration. The filtrate was concentrated using low-pressure distillation at 45°C and evaporated to dryness. We obtained 690±1 g of total extract solids from 4900 g powdered root (14.08%). To prepare the fractions, an aliquot of 300 g of the extract solids were re-suspended in 1400 mL of water/acetic acid (95:5) solution and further extracted with light petroleum ether (4 × 1000 mL) to separate the lipophilic compounds (ether fraction, 4.3% of total extract). The remaining aqueous part was treated with ammonia 25% and sequentially partitioned in chloroform and ethyl acetate (4 × 1000 mL for each of them). The solvent was removed under reduced pressure and the total alkaline residue (1.6%) was used for the experiments (alkaline fraction). All extracts were analyzed by thin layer chromatography (TLC). Plant total extract was prepared in 1% carboxymethylcellulose (CMC) in saline prior to animal testing. The alkaline and ether fractions were dissolved in saline containing 2% DMSO.

### Pharmacological studies

Male Swiss white mice, 20–25 g, were obtained from the Animal Care Facility (Faculty of Pharmacy, Tehran, Iran). The animals were housed six per cage in a temperature-controlled (22 ± 1°C) colony room. They were maintained in a 12h light/dark schedule with *ad libitum* food and water except during experimental procedures. All trials were carried out in the light phase. Subjects were experimentally naïve and each mouse was used only once. Animals were allowed 7 days to acclimatize to the laboratory environment including handling before testing began. All procedures were carried out in accordance with institutional guidelines for animal care and use. The protocol (No. 357) had been approved by the Committee of Ethics of the Faculty of Sciences of Tehran University.

Plant extracts and fractions were injected intraperitoneally (*i.p.*) in a single dose. Control groups received vehicle in saline (saline group) or diazepam 1 mg/kg, as a known anti-anxiety drug. The elevated plus maze consisted two open arms (6 × 30 × 2 cm) and two closed arms (6 × 30 × 10 cm), having an open roof, elevated 40 cm with a central platform of 8 × 8 cm. The test was performed 30 and 90 min after injection. Each mouse was placed in the central platform facing toward a closed arm and the cumulative time spent in open or closed arms was recorded for 5 min. The percent time spent was used as the measure of plus-maze performance. The ratio of percent time spent in open to closed arm (Ratio = % time spent in open arms/% time spent in closed arms) was indexed as the anxiolytic effect of various groups. Based on this calculation, when the animal had equal preference for open and closed arms at ratio = 1, this was an indicator of loss of anxiety. A ratio < 1 indicated that the animal avoided the open arm, indicating anxiety behavior. The entry time into each arm and the total entry time for each mouse were used as an index of activity for each animal. There were 4 mice in each group and the experiment was repeated 3 times, independently (n = 12).

### Isolation, purification, and structure elucidation

The alkaline fraction (2 g) was subjected to column chromatography (100 g silica gel) for clean-up and initial fractionation. Elution with 1200 mL of CHCl_3_/EtOAC (80:20) gave a fraction that contained major compounds of the chloroform extraction. This fraction was further purified by HPLC to yield compounds 1 and 2 as described below. Compound 3 eluted from the column with an Rf value greater than 1 and 2. This compound was further purified by preparative TLC by CHCl_3_/EtOAC (65:35) as the mobile phase.

For further purification, the CHCl_3_/EtOAC (80:20) fraction from column chromatography was subjected to preparative HPLC. Initially, a small amount of the fraction was injected into the analytical HPLC and the composition of the mobile phase was optimized by varying the percent of acetonitrile in phosphate buffer for separation of compounds 1 and 2. The best purification was obtained using the following conditions: acetonitrile/0.1 M phosphate buffer/glacial acetic acid (6:94:1 v/v/v), pH 4.15; flow rate: 1.2 mL/min; detector wavelength 300 nm. After optimizations using analytical HPLC, scale-up was performed for preparative separation. A flow rate of 14 mL/min was applied for preparative separation; other conditions were identical with the analytical method. The CHCl_3_/EtOAC (80:20) fraction was subjected to preparative HPLC and pure compounds 1 (32 mg) and 2 (12 mg) were separated collected in the mobile phase. The purity of these two compounds was re-checked by analytical HPLC and structure elucidation of the purified compounds was carried out by spectroscopic methods (MS, FTIR, ^1^H and ^13^C-NMR).

### Statistical analysis

The data obtained from independent pharmacological experiments were pooled and reported as mean ± SEM. The data were analyzed by one-way ANOVA followed by Tukey post hoc multiple comparison. P < 0.05 was considered significant.

## Results

### The anxiolytic effect of total extract in mice

**Figure 1:**
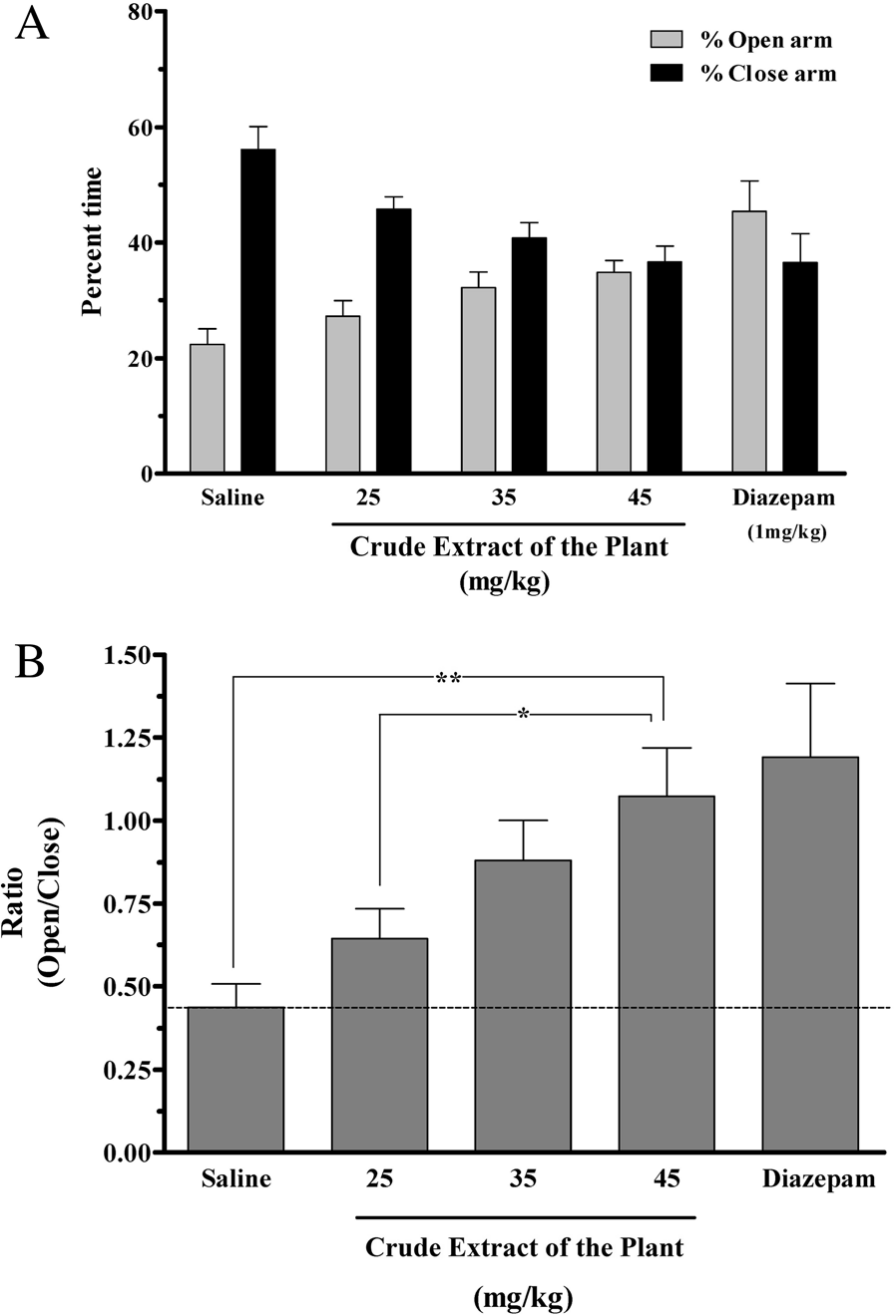
**Evaluation of the effects of a crude extract from *Biebersteinia multifida *roots on mouse behavior on an elevated plus-maze. A)** The percent time spent in each arm and **B)** The anxiolytic ratio as ratio of percent time spent in open arms to the close arms (Ratio = % time in open arms/% time in closed arms). The animals were injected 30 min prior to the test with saline (vehicle), diazepam (1 mg/kg) or different doses of crude extract. Data are presented as mean ± SE for each group (n = 12 in each group). The dotted line indicates the Ratio (open/close) of the vehicle (saline). *p value < 0.05, **p value < 0.01.

**Figure 2:**
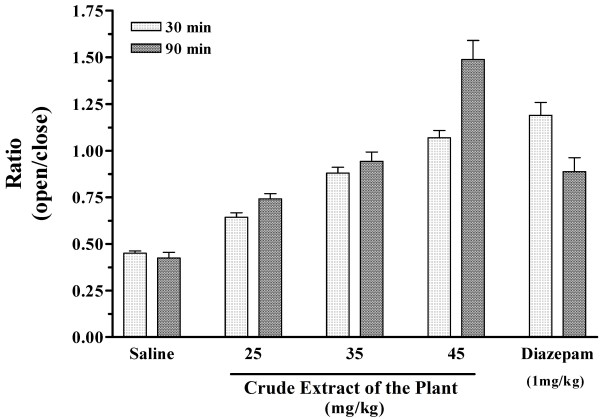
**The time dependency of the anxiolytic effect of total extract.** Effects were analyzed by performing the test at 30 min and 90 min after injection. The animals were injected 30 min prior to the plus maze test with saline (vehicle), diazepam (1 mg/kg) and different doses of crude extract of the plant. The ratio of percent time spent in open arms to close arms was calculated and plotted as mean ± SE for each group (n = 12 in each group).

In animals receiving the total extract of the roots and tested 30 min after injection, the time spent in open arm was prolonged compared to the animals that received saline (Figure 1). The effect was first seen at a dose of 25 mg extract/kg body weight and increased in a dose dependent manner to its maximum level at 45 mg/kg, where the animals spent equal time in the open and closed arms. The effect of 45 mg/kg of the total extract was comparable to that of diazepam at 1 mg/kg, where the mice showed a slightly more, but not significant, preference for the open arm (Figure 1A). At lower dose (10 mg/kg), the animals behaved similar to those given the saline treatment. At higher doses (50 and 75 mg/kg), the animals continued to show equal preference for the open and closed arms. The extract had very little effect on the activity of the animals at the doses used. As indicated in Table 1, the total entries into the closed and open arms are equal in all groups. In animals receiving 50 and 75 mg/kg of the extract, the entries were lower and they were mostly in sleep (Table 1). To compare the anxiolytic effect in various groups, we calculated the ratio of percent time spent in open arm to closed arm (see *Materials and Methods*) for each group (Figure 1B). The ratio = 1 indicates an equal preference for the open and the closed arms, representing loss of anxiety; and the ratio lower than 1 implies less preference for the open arm and an increase in anxiety behavior. This ratio in animals receiving 25 mg/kg of the total extract was higher (0.64±0.09) than in the saline group (0.44±0.07), indicating a trend toward preference for the open area although the difference was not statistically significant (Figure 1B). The anxiolytic ratio increased with increases in the dose of total extract and reached 1.07±0.15 in the mice that received 45 mg/kg of the total extract (p < 0.01 compared to saline), suggesting a lack of anxiety. This ratio was comparable to that obtained with diazepam (1.19±0.22, p < 0.01 compared to saline, Figure 1B). In order to evaluate the duration of anxiolytic effect, we also tested the effect of total extract 90 min after injection. As seen in Figure 2, the anxiolytic effect of the plant extract was sustained for 90 min. This anxiolytic ratio was even higher at 90 min after injection of 45 mg/kg of the total extract (p < 0.05 compared to 30 min, Figure 2). On the other hand, the effect of diazepam had been reduced considerably by 90 min. This observation suggests a longer duration of action for the extract compared to that of diazepam (Figure 2).

**Table 1:** **The effect of total extract of *Biebersteinia multifida *on the behavior of mice on an elevated plus-maze**

	**Total entry**		**Percent entry into open arms**	
**Treatment**	**30 min**	**90 min**	**30 min**	**90 min**
**Saline**	14.5 ± 1.5^a^	14.5 ± 0.9	41.2 ± 3.8	39.2 ± 4.3
**Diazepam (1 mg/kg)**	24.2 ± 2.7	18.3 ± 2.2	57.2 ± 3.55	44.2 ± 2.4
**Extract (25 mg/kg)**	15.7 ± 1.3	16.1 ± 1.3	41.2 ± 2.4	46.4 ± 2.2
**Extract (35 mg/kg)**	12.4 ± 1.3	14.3 ± 0.8	43.1 ± 1.7	44.2 ± 3.5
**Extract (45 mg/kg)**	16.4 ± 1.0	12.8 ± 1.4	50.5 ± 2.2	52.1 ± 2.8
**Extract (50 mg/kg)**	15.5 ± 3.6	10.0 ± 2.2	48.8 ± 1.9	44.0 ± 12.3
**Extract (75 mg/kg)**	8.2 ± 1.2	ND^b^	53.0 ± 6.2	ND

^a^The data are presented as mean ± SE (n = 12).

^b^*ND* not determined.

The table indicates the total entry and percent entry into open arms as an indicator of the activity of the animal. The entry to each arm was measured at 30 and 90 min after administration of the total extract.

### The anxiolytic effect of alkaline and ether fractions

**Figure 3:**
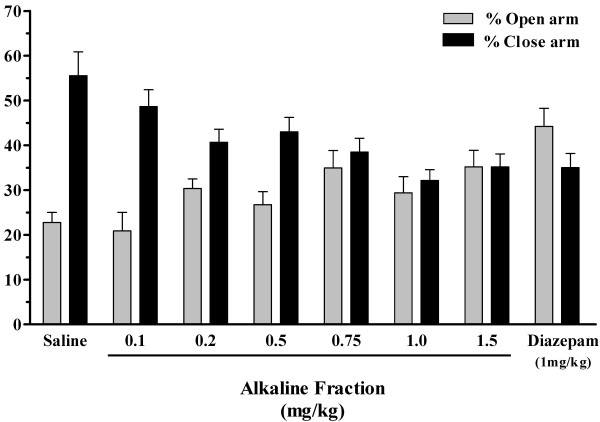
**The effect of an alkaline fraction of a *Biebersteinia multifida *root extract on the behavior of mice on an elevated plus-maze by the percent time spent in each arm.** The animals were injected 30 min prior to the test with saline (vehicle), diazepam (1 mg/kg) or different doses of alkaline fraction. Data are presented as mean ± SE for each group (n = 12 in each group).

**Figure 4:**
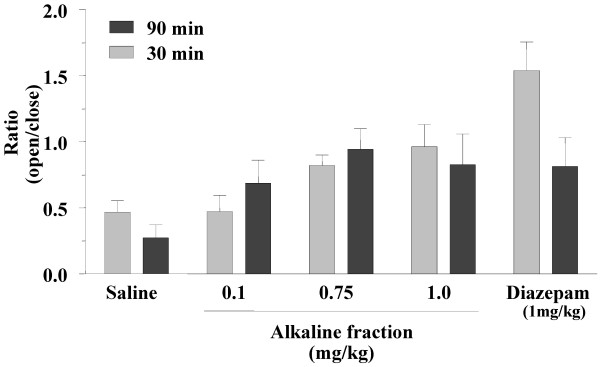
**The time dependency of the anxiolytic effect of an alkaline fraction from *Biebersteinia multifida *roots on mice.** The elevated plus-maze test was performed 30 min and 90 min after injection. The animals were injected 30 min prior to the test with saline (vehicle), diazepam (1 mg/kg) or different doses of alkaline fraction. The ratio of percent time spent in open arms to close arms was calculated and plotted as mean ± SE for each group (n = 12 in each group).

To further isolate the active compounds in *B. multifida,* the total root extract was fractionated as mentioned in *Materials and Methods*. Alkaloids of this plant may be responsible for its pharmacological activities [10]; thus we examined the anxiolytic activity of the alkaline and ether (lipophilic compounds) fractions of the root extract. Figure 3 shows the effects of various doses of the alkaline fraction on animal performance on elevated plus-maze 30 min after injection. An increase in tendency toward presence in the open arm was seen as the dose of the alkaline fraction was increased (Figure 3). The anxiolytic ratio calculated for these experiments indicated that this effect was dose dependent; it began at a dose of 0.2 mg/kg and the maximum effect was obtained at 1.0 mg/kg of the alkaline fraction. The activity of the animals indicated as total entries into the arms show no significant differences among the various groups (Table 2). We also studied the effect of the alkaline fraction 90 min after injection. As Figure 4 shows, unlike the diazepam treated group, the anxiolytic effect of alkaline fraction continued to be seen 90 min after injection, suggesting a sustained effect for this fraction.

**Table 2:** **The effect of alkaline fraction of *Biebersteinia multifida *on the behavior of mice on an elevated plus-maze**

	**Total entry**		**Percent entry to open arms**	
**Treatment**	**30 min**	**90 min**	**30 min**	**90 min**
**Saline**	14.2 ± 1.7^a^	8.8 ± 1.5	41.5 ± 3.3	39.9 ± 3.6
**Diazepam (1 mg/kg)**	32.2 ± 4.1	25.8 ± 6.2	60.5 ± 2.4	54.0 ± 6.4
**Alkaline fraction (0.1 mg/kg)**	19.2 ± 1.2	15.4 ± 1.7	42.3 ± 4.1	41.7 ± 6.9
**Alkaline fraction (0.2 mg/kg)**	14.8 ± 0.7	13.8 ± 1.4	48.8 ± 2.3	36.2 ± 2.5
**Alkaline fraction (0.5 mg/kg)**	13.8 ± 1.64	9.5 ± 1.3	41.0 ± 4.4	37.7 ± 4.9
**Alkaline fraction (0.75 mg/kg)**	16.5 ± 1.5	12.1 ± 1.1	51.2 ± 3.9	51.6 ± 3.6
**Alkaline fraction (1.0 mg/kg)**	23.0 ± 1.7	13.0 ± 4.3	48.4 ± 2.0	59.9 ± 3.1
**Alkaline fraction (1.5 mg/kg)**	18.2 ± 2.7	ND^b^	51.5 ± 2.7	ND

^a^The data are presented as Mean ± SE (n = 12).

^b^*ND* not determined.

The table indicates the total entry and percent entry into open arms as an indicator of the activity of the animal. The entry to each arm was measured at 30 and 90 min after administration of the alkaline fraction.

**Figure 5:**
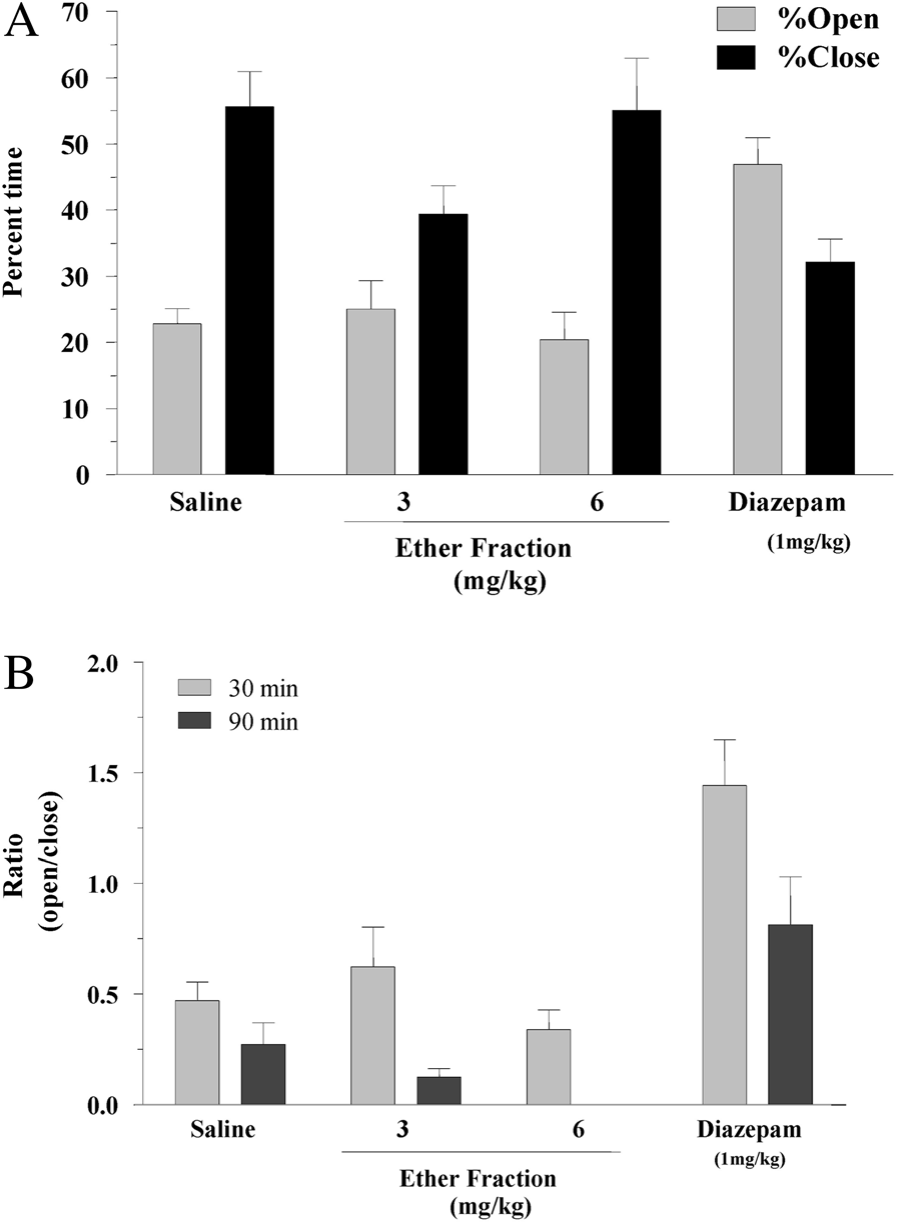
**The effect of an ether fraction of a *Biebersteinia multifida *root extract on the behavior of mice on an elevated plus-maze.** The animals were injected with ether fraction and the percent time spent in each arms of the plus maze test was plotted for each group. The ratio of time spent in open arms to close arms was calculated as an indicator of anxiety and fear of open and elevated area, and plotted for each group (Ratio = % time in open arms/% time in closed arms). The graph presents the results of three independent experiments (n =8 in each group).

We also tested the ether fraction of plant extract, which contained lipophilic compounds. At doses of 3 and 6 mg/kg of the ether fraction, the animals showed no significant anxiolytic effects compared to saline treated group (Figure 5A). The 3 and 6 mg of ether fraction were equivalent to 69.7 and 139.5 mg of total extract, respectively. The effect of the ether fraction was similar to saline and did not show any anxiolytic effect at either 30 or 90 min after injection (Figure 5B).

Collectively, the alkaline fraction produced an anxiolytic effect in a dose dependent manner starting at 0.2 mg/kg and reaching a maximum effect at 1.0 mg/kg. This effect was comparable to that seen for diazepam (1.0 mg/kg). Doses of 0.2 and 1.0 mg/kg of alkaloid fraction were equivalent to 12.5 and 62.5 mg/kg of the total root extract.

### Isolation, purification, and structure elucidation of chemicals present in the alkaline fraction

The alkaline fraction that showed the anxiolytic effect was subjected to different chromatography methods, as mentioned in *Materials and Methods*, which led to isolation and purification of three compounds.

**Figure 6:**
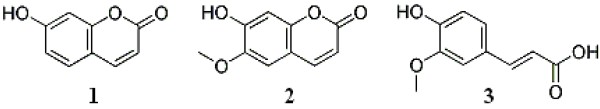
**Chemical structures of compounds isolated from a *Biebersteinia multifida *root extract, including umbelliferone (1), scopoletin (2) and ferulic acid (3).**

Structure elucidation of compounds 1, 2, and 3 was carried out by spectroscopic methods (MS, FTIR, ^1^H and ^13^C-NMR) and by comparison of acquired data with those reported in the literature [14-17]. Compounds 1, 2, and 3 were identified as umbelliferone, scopoletin, and ferulic acid, respectively (Figure 6, Additional file 1: Table S1 and Additional file 2: Figure S1, Additional file 3: Figure S2, Additional file 4: Figure S3, Additional file 5: Figure S4, Additional file 6: Figure S5, Additional file 7: Figure S6) as the major components of alkaloid fraction.

## Discussion

The anti-inflammatory and analgesic activities of *B. multifida* have been reported before [10]. We have employed a bio-guided fractionation to study the anxiolytic effect of the extracts. This approach has been successfully applied as a valuable strategy for the finding of new lead compounds in phytochemical studies [18].

The use of the elevated plus-maze to study animal anxiety-like behavior has been comprehensively studied and pharmacologically validated for rats [11] and mice [13]. In various studies, the open arm entry to total entry or open time ratio were used as an indicator of anxiety behavior in mice and rats [11,13]. Since locomotion is an important factor in the elevated plus-maze, the total entry time has been used as an indicator of activity [11,13]. In this study, the time spent in each arm, the open and closed time ratio, was used to evaluate the anxiety behavior, while the total entry time considered as an index of animal activity.

A bio-guided phytochemical analysis of *B. multifida* has not previously been reported. Although other reports have thus far identified a number of flavonoids, a few polysaccharides and one alkaloid (vasicinone) from root extract, in which vasicinone considered as the responsible molecule for observed pharmacological activities [5-7,9], there were no direct relation between the isolated compounds to traditional use of *B. multifida*. Thus, to identify the active anxiolytic components, the total extract is fractioned into alkaline and ether fractions and subjected to anxiety alleviation studies. The anxiolytic compounds act readily in alkaline fraction form while the ether fraction showed little anxiolytic effect. This bio-guided fractionation indicates that the active compounds are present in the alkaline fraction. Furthermore, the results suggest that the alkaloid extract has a sustained anxiolytic effect compared to that observed with diazepam. Diazepam is a known anxiolytic used as control in various pharmacological studies. In this study, our results indicate a strong anti anxiety effect in animals receiving diazepam (Figures 1 and 2). Interestingly, the anxiolytic effect of diazepam has been reduced considerably by 90 min (Figure 2). Thus compared to diazepam, the plant extract has a longer duration of action.

Before any further chromatographic studies, the effects of pure vasicinone -as the suspected active compound in the alkaloid fraction- on mouse performance is tested by the same experimental procedure as described above. The analysis of vasicinone, at doses as high as 10 mg/kg, had no significant effect on open time ratio (data not shown). Thus, the alkaline fraction is further characterized for the major components present which lead to isolation and structure elucidation of three compounds of coumarin derivatives including umbelliferone, scopoletin, and ferulic acid. However the presence of ferulic acid after alkaline extraction could be a consequence of ring opening of scopoletin after extraction.

According to previous reports, coumarin derivatives, including scopoletin and umbelliferone, are potent inhibitors of monoamine oxidases (MAOs) [19,20]. Scopoletin shows MAO inhibition activity in a dose-dependent fashion, with IC_50_ values of 19.4 μg/mL [19]. In addition, daphnoretin a bicoumarin of scopoletin and umbelliferone exhibits significant anxiolytic activity in EPM model [21-23]. It has been also suggested that coumarin derivatives interact with the benzodiazepine binding site of the GABA-A receptor [24]. This may explain the effects observed in the present study and may confirm at least a partial role of these coumarins anxiolytic effects of *B. multifida*.

In addition to the anxiolytic effect, scopoletin is also a known potent anti-inflammatory agent [25,26], which can explain, in part, the anti-inflammatory and anti-nociceptive effects of *B. multifida* extracts. The ability of low molecular weight substances to cross the blood–brain barrier could explain the reported central anti-nociceptive effect [10]. However, this needs further specific pure substance studies in validated pharmacological models.

In conclusion, for the first time, bio-guided fractionation of the root extract of *B. multifida* indicates significant sustained anxiolytic effects which led to isolation of three coumarin derivatives of those scopoletin and umbelliferone with known MAO inhibitory and anti-anxiety effects. These data contribute to evidence-based traditional medicines using root of *B. multifida* for anxiety disorders.

## Competing interests

The authors declare that they have no conflicts of interest.

## Authors’ contributions

HRM participated in the design of the study and carried out the phytochemical studies. MA carried out the structure elucidation. NG carried out the extraction, isolation and HPLC analysis and wrote the manuscript. FS carried out the animal studies and drafted the manuscript. RH participated in extraction and chromatography studies. MAF participated in the phytochemical analysis. ZT participated in experimental coordination and helped to draft the manuscript. MHG designed the study, performed the statistical analysis and finalized the manuscript. All authors read and approved the final manuscript.

## Supplementary Material

Additional file 1: Table S1 NMR chemical shift assignments of umbelliferone (1), scopoletin (2) and ferulic acid (3). Click here for file

Additional file 2: Figure S1 Mass spectrum of Umbelliferone. Click here for file

Additional file 3: Figure S2 FTIR spectrum of Umbelliferone. Click here for file

Additional file 4: Figure S3 Mass spectrum of Scopoletin. Click here for file

Additional file 5: Figure S4 FTIR spectrum of Scopoletin. Click here for file

Additional file 6: Figure S5 Mass spectrum of Ferulic acid. Click here for file

Additional file 7: Figure S6 FTIR spectrum of Ferulic acid. Click here for file

## References

[B1] Muellner AN Vassiliades DD Renner SS Placing Biebersteiniaceae, a herbaceous clade of Sapindales, in a temporal and geographic context Plant Syst Evol 2007 266 233 252 10.1007/s00606-007-0546-x

[B2] Amin G Popular Medicinal Plants of Iran 1991 1 Tehran: Research Deputy, Ministry of Health, Treatment and Medical Education

[B3] Amirghofran Z Medicinal plants as immunosuppressive agents in traditional Iranian medicine Iran J Immunol 2010 7 65 73 20574119 10.22034/iji.2010.17041

[B4] Aboutorabi H Ethnobotanic and phytochemical study of plants in Rouin region. PharmD Thesis 2001 Tehran: Tehran University of Medical Sciences

[B5] Arifkhodzhaev AO Rakhimov DA Polysaccharides of saponin-bearing plants. V. Structural investigation of glucans A, B, and C and their oligosaccharides from Biebersteinia multifida plants Chem Nat Compd 1994 30 655 660 10.1007/BF00630596

[B6] Greenham J Vassiliades DD Harborne JB Williams CA Eagles J Grayer RJ Veitch NC A distinctive flavonoid chemistry for the anomalous genus Biebersteinia Phytochemistry 2001 56 87 91 10.1016/S0031-9422(00)00355-1 11198823

[B7] Omurkamzinova VB Maurel ND Bikbulatova TN Flavonoids of Biebersteinia multifida Chem Nat Compd 1991 27 636 637 10.1007/BF00630376

[B8] Nabavi SF Ebrahimzadeh MA Nabavi SM Eslami B Dehpour A Antihemolytic and antioxidant activities of Biebersteinia multifida Eur Rev Med Pharmacol Sci 2010 14 823 830 21222368

[B9] Javidnia K Miri R Soltani M Khosravi AR Essential oil composition of biebersteinia multifida DC. (Biebersteiniaceae) from Iran J Essent Oil Res 2010 22 611 612 10.1080/10412905.2010.9700413

[B10] Farsam H Amanlou M Dehpour AR Jahaniani F Anti-inflammatory and analgesic activity of Biebersteinia multifida DC. root extract J Ethnopharmacol 2000 71 443 447 10.1016/S0378-8741(00)00174-4 10940581

[B11] Pellow S Chopin P File SE Briley M Validation of open: closed arm entries in an elevated plus-maze as a measure of anxiety in the rat J Neurosci Methods 1985 14 149 167 10.1016/0165-0270(85)90031-7 2864480

[B12] Rabbani M Sajjadi SE Jalali A Hydroalcohol extract and fractions of Stachys lavandulifolia vahl: effects on spontaneous motor activity and elevated plus-maze behaviour Phytother Res 2005 19 854 858 10.1002/ptr.1701 16261514

[B13] Lister RG The use of a plus-maze to measure anxiety in the mouse Psychopharmacology 1987 92 180 185 3110839 10.1007/BF00177912

[B14] Yan J Tong S Sheng L Lou J Preparative isolation and purification of two coumarins from Edgeworthia chrysantha Lindl by high speed countercurrent chromatography J Liq Chromatogr Related Technol 2006 29 1307 1315 10.1080/10826070600598969

[B15] Aplin RT Page CB The constituents of native umbelliferae. Part I. Coumarins from dill (Anetheum graveolens L.) J Chem Soc C 1967 2593 2596

[B16] Torres R Urbina F Morales C Modak B Delle Monache F Antioxidant properties of lignans and ferulic acid from the resinous exudate of Larrea nitida J Chil Chem Soc 2003 48 61 63

[B17] Dobhal MP Hasan AM Sharma MC Joshi BC Ferulic acid esters from Plumeria bicolor Phytochemistry 1999 51 319 321 10.1016/S0031-9422(99)00006-0

[B18] Pieters L Vlietinck AJ Bioguided isolation of pharmacologically active plant components, still a valuable strategy for the finding of new lead compounds? J Ethnopharmacol 2005 100 57 60 10.1016/j.jep.2005.05.029 15996842

[B19] Yun BS Lee IK Ryoo IJ Yoo ID Coumarins with monoamine oxidase inhibitory activity and antioxidative coumarino-lignans from Hibiscus syriacus J Nat Prod 2001 64 1238 1240 10.1021/np0100946 11575966

[B20] Seon HJ Xiang HH Seong SH Ji SH Ji HH Lee D Myung KL Jai SR Bang YH Monoamine oxidase inhibitory coumarins from the aerial parts of Dictamnus albus Arch Pharm Res 2006 29 1119 1124 10.1007/BF02969302 17225461

[B21] Herrera-Ruiz M Gonzalez-Carranza A Zamilpa A Jimenez-Ferrer E Huerta-Reyes M Navarro-Garcia VM The standardized extract of Loeselia mexicana possesses anxiolytic activity through the gamma-amino butyric acid mechanism J Ethnopharmacol 2011 138 261 267 10.1016/j.jep.2011.09.010 21979412

[B22] Navarro-García VM Herrera-Ruiz M Rojas G Zepeda LG Coumarin derivatives from Loeselia mexicana. Determination of the anxiolytic effect of daphnoretin on elevated plus-maze J Mex Chem Soc 2007 51 193 197

[B23] Kumar D Bhat ZA Kumar V Shah MY Coumarins from Angelica archangelica Linn. and their effects on anxiety-like behavior Prog Neuropsychopharmacol Biol Psychiatry 2013 40 180 186 22960104 10.1016/j.pnpbp.2012.08.004

[B24] Singhuber J Baburin I Ecker GF Kopp B Hering S Insights into structure–activity relationship of GABAA receptor modulating coumarins and furanocoumarins Eur J Pharmacol 2011 668 57 64 10.1016/j.ejphar.2011.06.034 21749864 PMC3196836

[B25] Moon PD Lee BH Jeong HJ An HJ Park SJ Kim HR Ko SG Um JY Hong SH Kim HM Use of scopoletin to inhibit the production of inflammatory cytokines through inhibition of the IκB/NF-κB signal cascade in the human mast cell line HMC-1 Eur J Pharmacol 2007 555 218 225 10.1016/j.ejphar.2006.10.021 17113069

[B26] Meotti FC Ardenghi JV Pretto JB Souza MM D’Ávila Moura J Cunha A Jr Soldi C Pizzolatti MG Santos ARS Antinociceptive properties of coumarins, steroid and dihydrostyryl-2- pyrones from Polygala sabulosa (Polygalaceae) in mice J Pharm Pharmacol 2006 58 107 112 10.1211/jpp.58.1.0013 16393470

