# Supplementary material for: Coumarin compounds of Biebersteinia multifida roots show potential anxiolytic effects in mice
Source: Daru. 2013 Jun 27;21(1):51. doi: 10.1186/2008-2231-21-51 (PMC3707806; doi:10.1186/2008-2231-21-51)
Supplement: Additional file 1: Table S1 — NMR chemical shift assignments of umbelliferone (1), scopoletin (2) and ferulic acid (3). [file 2008-2231-21-51-S1.docx]

**Coumarin compounds of *Biebersteinia multifida* roots show potential anxiolytic effects in mice**

Hamid Reza Monsef-Esfahani^1^, Mohsen Amini^2^, Navid Goodarzi^3,4^, Fatemeh Saiedmohammadi^1,5^, Reza Hajiaghaee^6^, Mohammad Ali Faramarzi^7^,Zahra Tofighi^1^, Mohammad Hossein Ghahremani^4,5,^*

*^1^Department of Pharmacognosy, Faculty of Pharmacy, Tehran University of Medical Sciences, Tehran, Iran; ^2^Department of Medicinal Chemistry, Faculty of Pharmacy, Tehran University of Medical Sciences, Tehran, Iran; ^3^Department of Pharmaceutics, Faculty of Pharmacy, Tehran University of Medical Sciences, Tehran, Iran; ^4^Nanotechnology Research Centre, Faculty of Pharmacy, Tehran University of Medical Sciences, Tehran, Iran; ^5^Department of Pharmacology and Toxicology, Faculty of Pharmacy, Tehran University of Medical Sciences, Tehran, Iran; ^6^Department of Pharmacognosy and Pharmaceutics, Institute of Medicinal Plants, ACECR, Tehran, Iran; ^7^Department of Pharmaceutical Biotechnology, Faculty of Pharmacy& Biotechnology Research Center, Tehran University of Medical Sciences, Tehran, Iran*

***Corresponding author:** Mohammad Hossein Ghahremani

E-mail addresses: HRM: [monsefes@tums.ac.ir](mailto:monsefes@tums.ac.ir); MA: [moamini@tums.ac.ir](mailto:moamini@tums.ac.ir); NG: [goodarzi_n@razi.tums.ac.ir](mailto:goodarzi_n@razi.tums.ac.ir); FS: fsmohammadi@gmail.com; RH: [rhajiaghaee@yahoo.com](mailto:rhajiaghaee@yahoo.com); MAF: [faramarz@tums.ac.ir](mailto:faramarz@tums.ac.ir); ZT: [ztofighi@razi.tums.ac.ir](mailto:ztofighi@razi.tums.ac.ir); MHG: [mhghahremani@tums.ac.ir](mailto:mhghahremani@tums.ac.ir)

**Table 1S.** NMR chemical shift assignments of umbelliferone (1), scopoletin (2) and ferulic acid (3)

|  | **Compound 1**  **(in DMSO-*d_6_*)** | | **Compound 2**  **(in DMSO-*d_6_*)** | | **Compound 3**  **(in CDCl_3_)** | |
| --- | --- | --- | --- | --- | --- | --- |
|  | **^13^C (δ)** | **^1^H (δ)** | **^13^C (δ)** | **^1^H (δ)** | **^13^C (δ)** | **^1^H (δ)** |
| **1** |  |  |  |  | 127.04 |  |
| **2** | 161.53 |  | 161.88 |  | 109.26 | 7.05, s |
| **3** | 111.40 | 6.15, d, J=9.5 Hz | 110.22 | 6.0, d, J=9 Hz | 146.74 |  |
| **4** | 143.90 | 7.65, d, J=9.5 Hz | 144.27 | 7.6, d, J=9 Hz | 147.86 |  |
| **4a** | 111.29 |  | 109.45 |  |  |  |
| **5** | 128.88 | 7.31, d, J=7.5 Hz | 108.27 | 6.8, s | 115.67 | 6.9, d, J=8 Hz |
| **6** | 113.53 | 6.81, d, J=7.5 Hz | 145.80 |  | 123.08 | 7.1, d, J=8 Hz |
| **7** | 161.86 |  | 150.85 |  | 144.68 | 7.63, d, J=15.6Hz |
| **8** | 102.93 | 6.80 s | 103.41 | 6.6, s | 114.68 | 6.3, d, J=15.6 Hz |
| **8a** | 155.83 |  | 146.39 |  |  |  |
| **9** |  |  |  |  | 167.40 |  |
| **OMe** |  |  | 56.11 | 3.88, s | 55.96 | 3.9, s |
|  |  |  |  |  |  | 5.94, br, OH |

**FTIR and MS spectral data of compounds**

Umbelliferone (1): FTIR (KBr) 3165, 1705, 1685, 1603, 1562, 1403, 1326, 1137, 835 cm^-1^; EI-MS m/z (%), 162.5 (43) (M^+^, C_9_H_6_O_3_), 134.5 (49), 105 (12), 84.4 (100), 66.5 (83).

Scopoletin (2): FTIR (KBr) 3472, 1705, 1577, 1403, 1132, 1034, 1004 cm^-1^;EI-MS m/z (%), 192.8 (32) (M^+^, C_10_H_8_O_4_), 177 (18), 163 (12), 149.6 (24), 121 (16), 84.7 (48), 66.4 (100).

Ferulic acid (3): FTIR (KBr) 3538, 3013, 2915, 2846, 1698, 1595, 1512, 1458 cm^-1^; EI-MS m/z (%), 194.5 (100) (M^+^, C_10_H_10_O_4_), 177.3 (94), 150.9 (37), 137.7 (52), 83.8 (28), 58.1 (38).

**Figure Captions**

Figure 1S- Mass spectrum of Umbelliferone

Figure 2S- FTIR spectrum of Umbelliferone

Figure 3S- Mass spectrum of Scopoletin

Figure 4S- FTIR spectrum of Scopoletin

Figure 5S- Mass spectrum of Ferulic acid

Figure 6S- FTIR spectrum of Ferulic acid
