# Supplementary figures and images for: Coumarin compounds of Biebersteinia multifida roots show potential anxiolytic effects in mice
Source: Daru. 2013 Jun 27;21(1):51. doi: 10.1186/2008-2231-21-51 (PMC3707806; doi:10.1186/2008-2231-21-51)

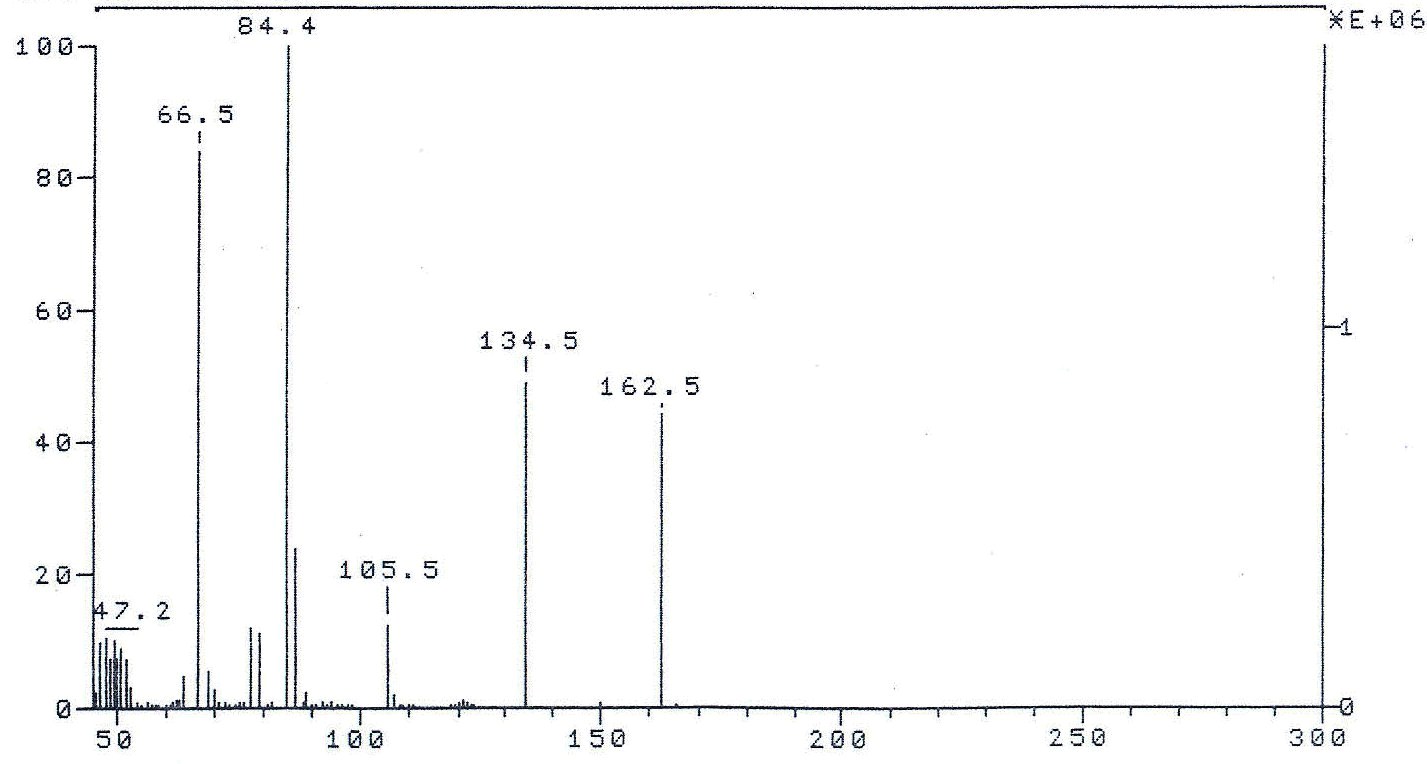

Supplement: Additional file 2: Figure S1 — Mass spectrum of Umbelliferone. [file 2008-2231-21-51-S2.jpeg]

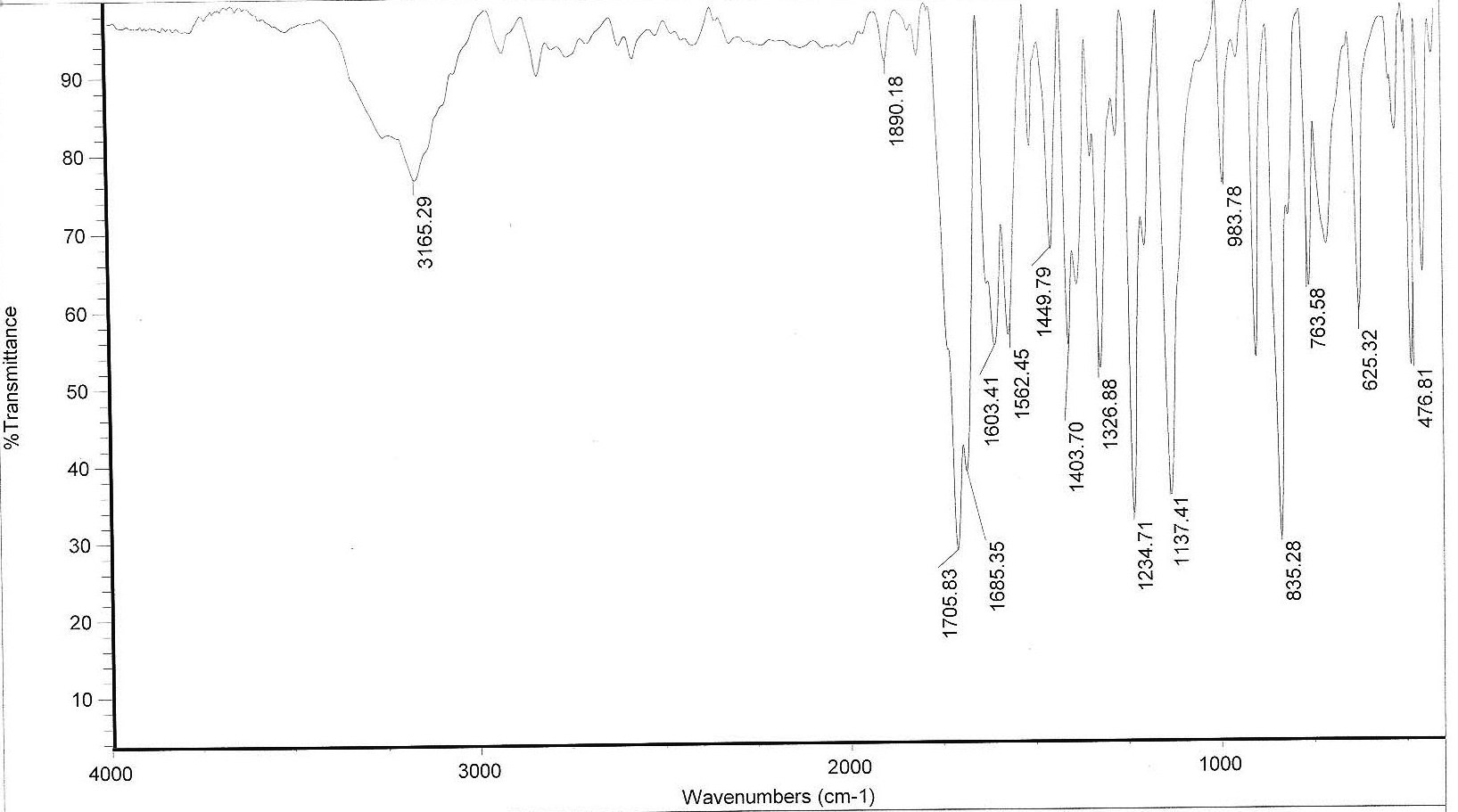

Supplement: Additional file 3: Figure S2 — FTIR spectrum of Umbelliferone. [file 2008-2231-21-51-S3.jpeg]

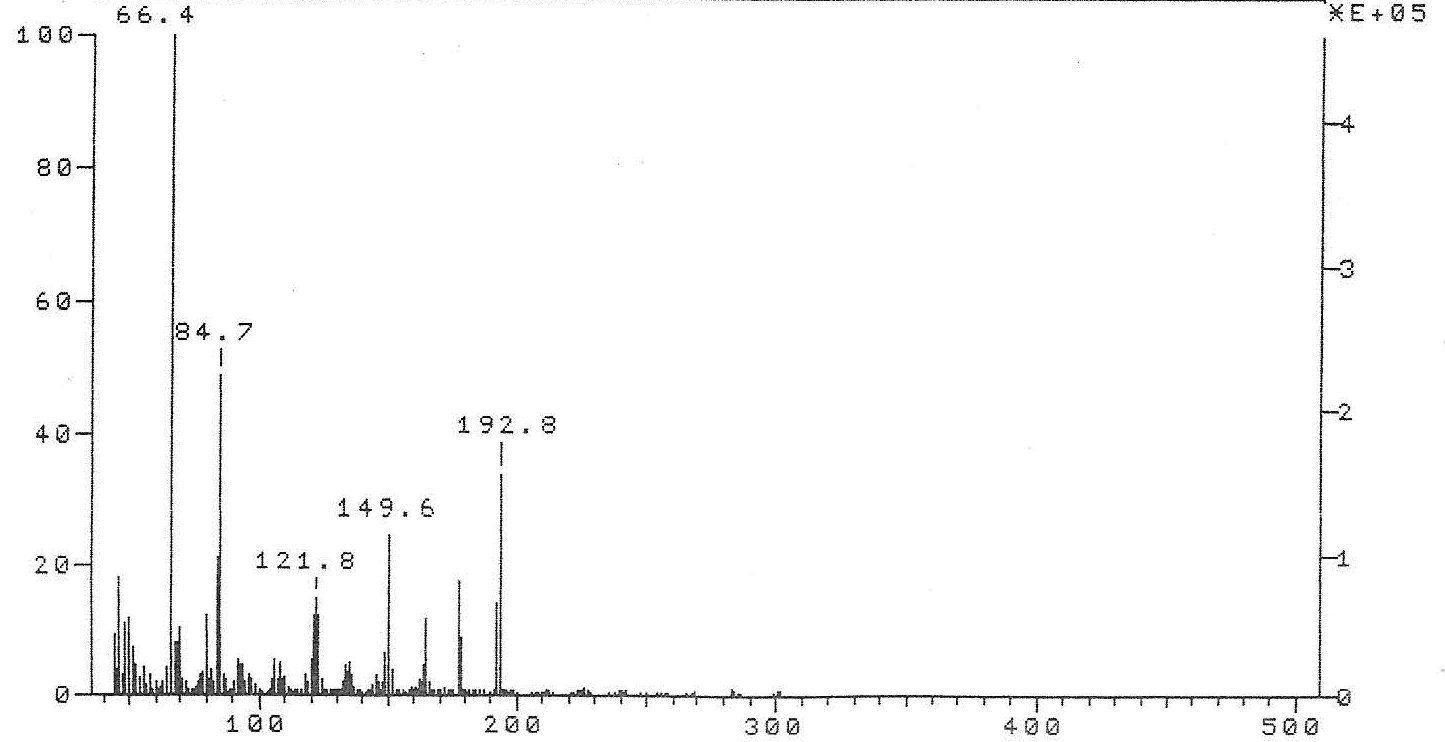

Supplement: Additional file 4: Figure S3 — Mass spectrum of Scopoletin. [file 2008-2231-21-51-S4.jpeg]

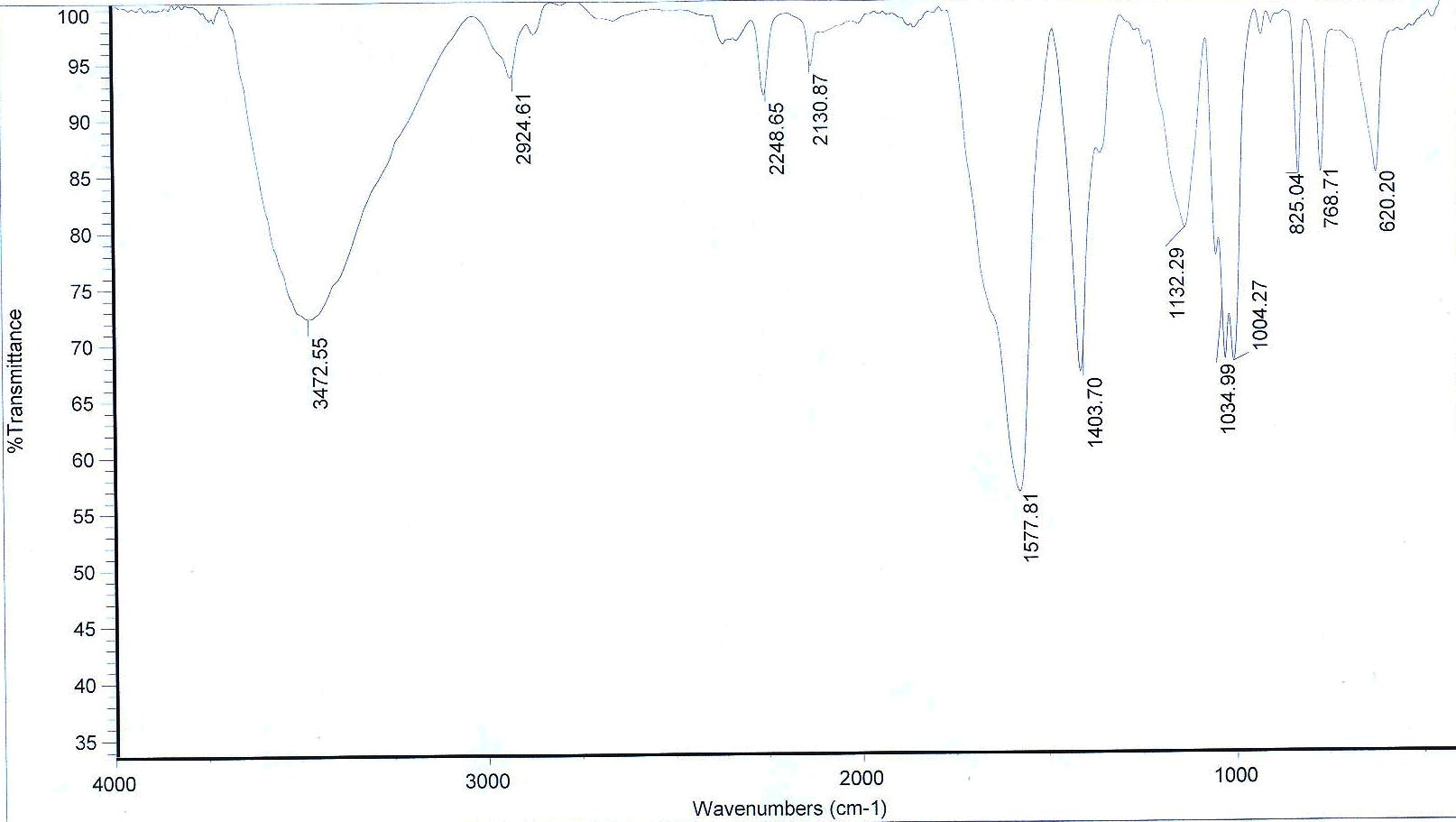

Supplement: Additional file 5: Figure S4 — FTIR spectrum of Scopoletin. [file 2008-2231-21-51-S5.jpeg]

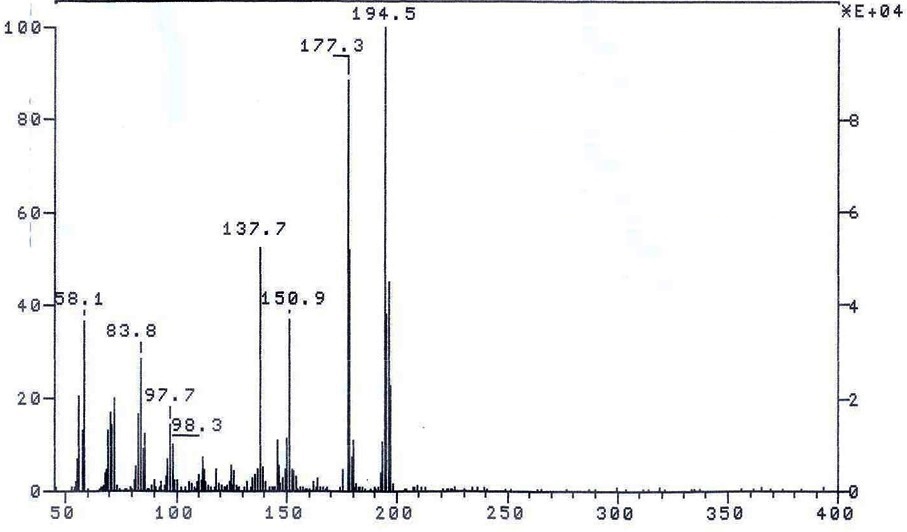

Supplement: Additional file 6: Figure S5 — Mass spectrum of Ferulic acid. [file 2008-2231-21-51-S6.jpeg]

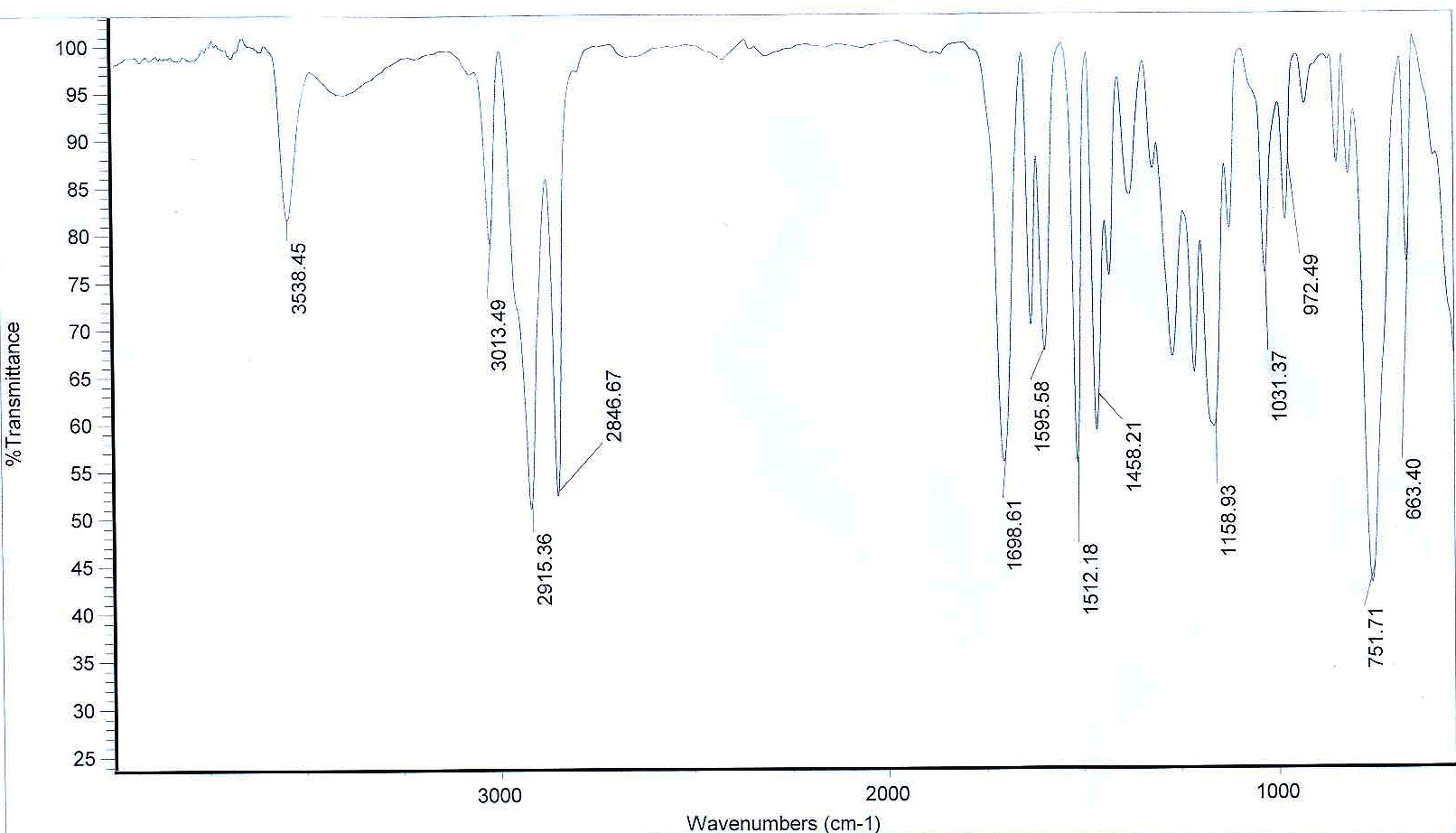

Supplement: Additional file 7: Figure S6 — FTIR spectrum of Ferulic acid. [file 2008-2231-21-51-S7.jpeg]
